# Measuring influencing factors affecting mortality rates during the COVID-19 pandemic

**DOI:** 10.1080/16549716.2024.2428067

**Published:** 2024-11-12

**Authors:** Jih-Shong Wu, Kuo-Kuang Huang

**Affiliations:** a College of General Education, Chihlee University of Technology, New Taipei City, Taiwan; b Department of International Trade, Chihlee University of Technology, New Taipei City, Taiwan

**Keywords:** COVID-19, infection, mortality, inefficiency, stochastic Frontier analysis

## Abstract

**Background:**

The COVID-19 pandemic has revealed clear deficiencies in global public health policies and healthcare systems when confronted with the emergence of a novel and deadly infectious disease.

**Objectives:**

With 4 years elapsed since the onset of the pandemic, ample data now exist to analyse the associations between the implementation of diverse public health policies, sociodemographic factors and COVID-19 mortality rates.

**Methods:**

This study utilised the dataset compiled by ‘Our World in Data’ spanning the period of the COVID-19 pandemic from 2020 to 2022. Stochastic frontier analysis was employed to assess the influencing factors and their relationship with mortality rates resulting from COVID-19 infections across 156 countries or regions.

**Results:**

This study yielded several key findings: (1) There remains a 33% margin for improvement in the global mortality rate concerning the COVID-19 pandemic; (2) During the initial stage of the pandemic, when an effective vaccine was not yet available, implementing public health control policies could reduce both the infection and mortality rates; (3) Areas characterised by higher population densities, a greater proportion of individuals aged 65 and over, and elevated prevalence rates of diabetes demonstrated higher mortality rates; and (4) Increasing vaccination coverage emerged as an effective strategy for reducing mortality rates.

**Conclusions:**

As our understanding of the COVID-19 virus improves, global economies and social interactions have gradually returned to normality. It is anticipated that the findings of this study can serve as a valuable reference in combating potential future pandemics caused by unknown viruses.

## Paper context

- **Main findings:** The initial social distancing and lockdown measures, and use of masks as a preventive measure against the spread of COVID-19 disease, among many public health policies, have proven effective.
- **Added knowledge:** This study uncovered several factors influencing COVID-19 mortality, including the infection rate, population density, proportion of elderly individuals, intensity of public health control measures, presence of cardiovascular disease and diabetes and the cumulative fully vaccinated rate.
- **Global health impact for policy and action:** The results of this study are anticipated to serve as valuable references and effective guides for policymakers and public health authorities worldwide, especially in the event of future unknown deadly epidemics or public health crises.

## Background

A highly contagious virus known as SARS-CoV-2 was first identified in Wuhan, China, in December 2019, leading to the emergence of a disease named ‘COVID-19’ [1,2]. Subsequently, within a few weeks, COVID-19 became the focus of global headlines, marked by a notable surge in daily confirmed cases and fatalities [3,4]. Recognising its severity, the World Health Organization (WHO) declared COVID-19 a Public Health Emergency of International Concern on 30 January 2020, officially designating it as a pandemic on 11 March 2020 [5]. Subsequently, the COVID-19 public health emergency was lifted in May 2023 [6]. By the end of January 2024, over 700 million confirmed cases and over 69 million deaths had been recorded [7].

It has now been over 4 years since the onset of the COVID-19 pandemic. In response, countries worldwide have implemented numerous social policies and public health measures to mitigate the virus’ spread and minimise fatalities. Several studies have investigated the associations between individual behaviours, sanitary policy measures, sociodemographic factors, and COVID-19 morbidity and mortality rates (MRs) [8–10]. The majority of countries appeared ill-prepared to confront a pandemic of COVID-19’s magnitude, particularly during the initial stages when effective vaccination was unavailable. Nearly all resorted to social distancing and lockdown measures to curb transmission. In 2020, during the fight against the pandemic, Kyrgyzstan implemented a partial lockdown, while Kazakhstan enforced a full lockdown. Both measures were effective in reducing the growth rate of new cases, and their results were comparable [11]. However, these measures had a significant economic impact, leading to stagnation and a rise in the cost-of-living, with repercussions on people’s livelihoods and overall economic stability; these in turn affected their health and well-being [12–14]. Despite the economic toll, initial social distancing and lockdown measures, among many public health policies, have proven effective. These measures included remote work, reduced transportation usage, and limits on gatherings or activities of more than 50 people [15–17]. Both the US Centers for Disease Control and Prevention (CDC) and the WHO guidelines suggested the use of masks as a preventive measure against the spread of COVID-19 disease [18]. Based on epidemiological data, the implementation of universal mask mandates in Japan, Hong Kong, Singapore, South Korea and Taiwan has proven most effective in mitigating the spread of COVID-19 disease during the early stages [19]. Several studies indicate a positive correlation between population density and COVID-19 mortality, as higher population density facilitates virus transmission through close interpersonal contact, leading to increased transmission possibilities in densely populated areas [20–23]. Some studies suggest that clinical evidence increasingly links the risk of death from COVID-19 to age, with MRs significantly higher among individuals over 60 years old, and mortality risk increasing with age [17,24–27]. The US CDC [28] similarly identifies age as the most significant risk factor for severe COVID-19 outcomes, with a notable increase in mortality risk as age increases, particularly among those aged 65 and older. Furthermore, numerous studies indicate that individuals with cardiovascular disease and diabetes may have a heightened susceptibility to SARS-CoV-2 infection, placing them at increased risk of severe COVID-19 and mortality [29–33].

On 5 January 2021, WHO’s Strategic Advisory Group of Experts on Immunization convened a meeting and approved the Pfizer/BioNTech vaccine, which became the first vaccine to receive an emergency use authorisation for COVID-19 issued by WHO [5]. Recent research and observations have demonstrated that COVID-19 vaccines are the most effective and economical method to reduce and control SARS-CoV-2 infection. However, achieving herd immunity remains essential for ending the COVID-19 pandemic [34]. Moreover, complete vaccination against COVID-19 has demonstrated several benefits, including a reduction in the number of infected individuals across all age groups, lower hospitalisation rates or shortened hospital stays, decreased risk of ICU admission, and a notable decrease in mortality among the elderly population [3,35,36]. Nevertheless, it is essential to recognise that the risk of severe breakthrough COVID-19 infections after vaccination persists, especially among populations with a higher risk of critical illness [37,38].

Throughout the COVID-19 pandemic, numerous studies worldwide have investigated the impact and effectiveness of various treatment methods both before and after the confirmation of SARS-CoV-2 infection. However, only a handful of studies have focused on short-term assessments, spanning from a few months to a year, examining the efficiency and influencing factors in the global fight against the COVID-19 pandemic [39]. Four years after the onset of the COVID-19 outbreak, in January 2024, Tedros Adhanom Ghebreyesus, Director-General of the WHO, issued a warning about ‘Disease X’ to ensure global preparedness for the next wave of an unknown deadly virus pandemic [40].

This study differs from many short-term studies conducted in recent years, spanning from a few months to a year, which mainly focused on exploring the associations between the mortality of confirmed cases caused by SARS-CoV-2 and the risk factors of public health policies in a single country or region. Instead, this study integrated and quantified big data from countries and autonomous regions worldwide during the COVID-19 pandemic from 2020 to 2022. Additionally, this study employed a two-stage approach to model the data before and after effective vaccination began in 2021. Utilising stochastic frontier analysis (SFA), this study constructed a mathematical model over time to investigate the correlation between mortality of confirmed cases and personal, social and public health policy influencing factors during the COVID-19 pandemic. This study also attempted to identify factors associated with the mortality of confirmed cases and factors that ultimately reduce the MR of COVID-19. The results of this study are anticipated to serve as valuable references and effective guides for policymakers and public health authorities worldwide, especially in the event of future unknown deadly epidemics or public health crises.

## Methodology

### SFA method

Efficiency evaluation serves to assess the operational performance of a decision-making unit and to identify operational areas for improvement within that unit. Efficiency entails measuring the optimal utilisation of inputs and outputs with the objective of maximising output or minimising costs [2,41]. The SFA is a methodology initially proposed by Aigner et al. [42] and Meeusen and van den Broeck [43]. It revolves around establishing a production efficiency frontier by connecting the most efficient combinations of input and output points for the enterprise under evaluation. It is important to note that not all enterprises are deemed efficient; only those situated at the efficiency frontier production point are considered technically efficient. In this study, we employed the Cobb–Douglas stochastic frontier production function model proposed by Battese and Coelli [44] for analysing continuous intertemporal data. Moreover, recognising the influence of various external environmental factors on the MR of COVID-19, exogenous variables were incorporated into the analysis to explore the influencing factors of global MRs during the COVID-19 pandemic and their role in contributing to these inefficiencies. The Cobb–Douglas stochastic frontier model is represented by the following equations [45]:

(1) $$\ln Y_{\mathrm{it}}=\beta_{0}+\underset{j}{\sum }\beta_{j}\ln X_{\mathrm{it}}+V_{\mathrm{it}}-U_{\mathrm{it}}\;,\;i=1,\cdots N,t=1,\cdots T$$

(2) $$U_{\mathrm{it}}=\delta_{0}+\underset{j}{\sum }\delta_{j}\ln Z_{\mathrm{it}}+\cdots +\varepsilon_{\mathrm{it}}$$

where *Y*_*it*_ represents the appropriate type of output of the *i-th* manufacturer at time *t*; *X*_*it*_ represents the *i-th* vendor input at time *t*; *β* represents the individual input coefficient of the production function; *Z*_*it*_ is the exogenous variable; and *V*_*it*_ represents the symmetric interference term of random variation of the production function. Here, *V*_*it*_ *∼ iddN(0, σ*^*2*^_*v*_) and *U*_*it*_ are independent of each other. *U*_*it*_ is the inefficiency factor of manufacturer *i* at phase t. It represents the degree of production technology inefficiency and is the only nonnegative random variable. Finally, *δ*_*0*_ is the intercept term, and *ɛ*_*it*_ is a random error and a nonnegative truncated normal distribution [45].

### Samples and data sources

The samples and data utilised in this study were sourced from Our World in Data [46], a platform that was updated daily and maintained throughout the duration of the COVID-19 pandemic (2020–2022). The dataset pipeline was established through a combination of various channels, each operating independently. These data sources include contributions from the Our World in Data team, the WHO, and international organisations such as the United Nations, the World Bank, the Organization for Economic Cooperation and Development, and the Institute for Health Metrics and Evaluation, among others. Our World in Data primarily oversees the collection, processing and publication of data related to vaccination, testing and hospitalisation. However, the conversion and release of information regarding the number of cases and deaths are managed by the Johns Hopkins Coronavirus Resource Center.

This study aggregated data pertaining to the COVID-19 pandemic across multiple countries and autonomous regions worldwide from 2020 to 2022, utilising the COVID-19 dataset provided by Our World in Data [46]. Following the exclusion of countries and regions with incomplete data, a total of 156 countries and autonomous regions were included in the analysis. Table 1 presents a comprehensive overview of the compiled data.

**Table 1.** 156 countries and autonomous regions.

No.	Location	No.	Location	No.	Location	No.	Location	No.	Location	No.	Location	No.	Location
1	Afghanistan	24	Burundi	47	El Salvador	70	Iraq	93	Mauritius	116	Poland	139	Tajikistan
2	Albania	25	Cambodia	48	Estonia	71	Ireland	94	Mexico	117	Portugal	140	Tanzania
3	Algeria	26	Cameroon	49	Eswatini	72	Israel	95	Moldova	118	Qatar	141	Thailand
4	Angola	27	Canada	50	Ethiopia	73	Italy	96	Mongolia	119	Romania	142	Timor-Leste
5	Argentina	28	Cabo Verde	51	Fiji	74	Jamaica	97	Morocco	120	Russian	143	Togo
6	Australia	29	Central African	52	Finland	75	Japan	98	Mozambique	121	Rwanda	144	Trinidad
7	Austria	30	Chad	53	France	76	Jordan	99	Myanmar	122	Saudi Arabia	145	Tunisia
8	Azerbaijan	31	Chile	54	Gabon	77	Kazakhstan	100	Namibia	123	Senegal	146	Türkiye
9	Bahamas	32	China	55	Gambia	78	Kenya	101	Nepal	124	Serbia	147	Uganda
10	Bahrain	33	Colombia	56	Georgia	79	Kuwait	102	Netherlands	125	Seychelles	148	UAE
11	Bangladesh	34	Congo	57	Germany	80	Lao	103	New Zealand	126	Sierra Leone	149	UK
12	Barbados	35	Costa Rica	58	Ghana	81	Latvia	104	Nicaragua	127	Singapore	150	USA
13	Belarus	36	Côte d’Ivoire	59	Greece	82	Lebanon	105	Niger	128	Slovakia	151	Uruguay
14	Belgium	37	Croatia	60	Guatemala	83	Lesotho	106	Nigeria	129	Slovenia	152	Venezuela
15	Belize	38	Cuba	61	Guinea	84	Liberia	107	Norway	130	Solomon Islands	153	Viet Nam
16	Benin	39	Cyprus	62	Guyana	85	Libya	108	Oman	131	South Africa	154	Yemen
17	Bhutan	40	Czech	63	Haiti	86	Lithuania	109	Pakistan	132	South Korea	155	Zambia
18	Bolivia	41	Democratic	64	Honduras	87	Madagascar	110	Palestine	133	Spain	156	Zimbabwe
19	Botswana	42	Denmark	65	Hungary	88	Malawi	111	Panama	134	Sri Lanka		
20	Brazil	43	Djibouti	66	Iceland	89	Malaysia	112	Papua New Guinea	135	Sudan		
21	Brunei	44	Dominican	67	India	90	Mali	113	Paraguay	136	Suriname		
22	Bulgaria	45	Ecuador	68	Indonesia	91	Malta	114	Peru	137	Sweden		
23	Burkina Faso	46	Egypt	69	Iran	92	Mauritania	115	Philippines	138	Switzerland		

### Variables and empirical model

This study examined the severity of disease and mortality risks associated with the COVID-19 pandemic spanning from the beginning of 2020 to the end of 2022. Inputs, outputs, and exogenous variables were carefully selected with a focus on characteristics aimed at reducing MRs, to explore the influencing factors and effectiveness of global efforts in managing SARS-CoV-2 infection. The study outcomes are expected to offer valuable insights and practical applications across diverse sectors. Additionally, aligning with ongoing efforts to combat and mitigate COVID-19 positivity and MRs, the findings of this study are anticipated to help prepare for potential future waves of unknown deadly virus pandemics and offer guidance to prevent or reduce their impact.

This study compiled data spanning from January 2020 to December 2022 from the COVID-19 dataset provided by Our World in Data [46]. The MR of COVID-19 was utilised as the undesirable output. To clarify, the MR is calculated as the total deaths divided by the total cases of COVID-19 (MR = total deaths/total cases), representing the cumulative MR of COVID-19 infection in a given country or region each year. Here, ‘total deaths’ refers to the overall number of deaths attributed to COVID-19, while ‘total cases’ denotes the total confirmed cases of COVID-19.

The WHO suggests that measuring excess mortality is essential for understanding the impact of the pandemic. Trends in MRs can provide valuable insights for decision-makers, guiding policies to effectively reduce mortality rates and prevent future crises. However, in many countries, limited investment in data systems often obscures the true extent of excess deaths [2,47]. The MR estimates are based on the number of deaths relative to the number of confirmed cases of infection, which does not reflect the actual death rate. Asymptomatic cases or patients with very mild symptoms may not be tested and therefore remain unidentified. Such cases cannot be included in the estimation of the actual MR, as the estimates pertain only to clinically apparent COVID-19 cases [48]. There is indeed a discrepancy between the MR and the excess MR or the actual MR, resulting in slight differences in the data. Additionally, there is no standardised method or measure for estimating MRs across countries, resulting in significant variation, which may also introduce inaccuracies in the analysis results of this study. This study utilised the MR as an output of a function to examine whether input factors – such as population density, proportion of elderly population, public health control measures, high-risk chronic diseases, and vaccination rates – influenced higher mortality rates. The purpose was to provide a reference for future responses to unknown viruses. Consequently, there was indeed a discrepancy between the MRs and the excess mortality figures used in this study. Although real data were not available, the findings of this study can still serve as a useful reference for policy formulation when addressing unknown viruses in the future.

In this study, the input items encompassed the following: infection rate (IR), calculated as the total cases divided by the population (IR = total cases/population), representing the annual cumulative COVID-19 infection rate; population density (PD); and the proportion of the population aged 65 years and older (65Y). The aforementioned undesirable output and input items were incorporated into equation (1), from which equation (3) was derived.

(3) $$\ln\left(MR_{\mathrm{it}}\right)=\beta_{0}+\beta_{1}\ln(IR_{\mathrm{it}})+\beta_{2}\ln\left(PD_{\mathrm{it}}\right)+\beta_{3}\ln\left(65Y_{\mathrm{it}}\right)+V_{\mathrm{it}}-U_{\mathrm{it}}$$

This study divided the analysis of exogenous variables into two distinct phases. In the initial phase (2020), the exogenous variables comprised the stringency index (SI), cardiovascular death rate (CDR), and diabetes prevalence (DP). The main difference between the two phases is that during the second phase (2021–2022), most countries and regions globally acquired COVID-19 vaccines and commenced vaccination campaigns in 2021. Therefore, an additional exogenous variable introduced in the second phase was the cumulative fully vaccinated rate (FVR). Upon incorporating the exogenous variables from both phases into equation (2), equations (4) and (5) were derived. For the estimation of equations (3) and (4), as well as equations (3) and (5), the computational software utilised in this study was Frontier Version 4.1, graciously provided by Coelli [49] at no cost.

(4) $$U_{\mathrm{it}}=\delta_{0}+\delta_{1}\mathrm{lnS}I_{\mathrm{jt}}+\delta_{2}\mathrm{lnCD}R_{\mathrm{jt}}+\delta_{3}\mathrm{lnD}P_{\mathrm{jt}}+\cdots +\varepsilon_{\mathrm{it}}$$

(5) $$U_{\mathrm{it}}=\delta_{0}+\delta_{1}\mathrm{lnS}I_{\mathrm{jt}}+\delta_{2}\mathrm{lnCD}R_{\mathrm{jt}}+\delta_{3}\mathrm{lnD}P_{\mathrm{jt}}+\delta_{4}\mathrm{lnFV}R_{\mathrm{jt}}+\cdots +\varepsilon_{\mathrm{it}}$$

This study constructed a Cobb–Douglas stochastic frontier model to assess the relationship between MR inefficiency and influencing factors during the COVID-19 pandemic across 156 countries and autonomous regions worldwide. The inefficiency value, ranging from 0 to 1, indicates the overall inefficiency in controlling COVID-19 mortality, with a higher value signifying greater mortality and poorer overall pandemic control efficiency, thereby indicating inefficiency in the region. Table 2 provides a detailed description and statistical data of the variables analysed in this study.

**Table 2.** Descriptive statistics of variables.

Items	Variables	Samples	Mean	Std.	Min.	Max.	Median
Output item	Y= MR (%)	468	1.4685	2.1733	0.0012	29.0814	0.9470
Input item	X1= infection rate (%)	468	9.5509	16.5687	0.0005	88.4915	2.7972
	X2= population density, number of people divided by land area, measured in square kilometres, most recent year available.	468	200.5205	667.2255	1.9800	7915.7310	80.8190
	X3= 65 years and older (%), share of the population that is 65 years and older, most recent year available.	468	8.7384	6.3483	1.1440	27.0490	6.2175
Exogenous Variable	Z1= stringency index, Government Response Stringency Index: composite measure based on 9 response indicators including school closures, workplace closures, and travel bans, rescaled to a value from 0 to 100 (100 = strictest response).	468	44.6186	29.3232	7.6400	550.5000	46.0450
	Z2= cardiovascular death rate (%), Death rate from cardiovascular disease in 2017.	468	0.2530	0.1133	0.0794	0.5970	0.2393
	Z3= diabetes prevalence (%), Diabetes prevalence (% of population aged 20 to 79) in 2017.	468	7.6308	4.0108	0.9900	22.0200	6.9100
	Z4= cumulative fully vaccinated rate (%), Total number of people who received all doses prescribed by the initial/population) × 100%.	312	49.1334	27.0850	0.0274	105.7754	52.4637

### Empirical model discussion

SFA models estimate two main components: technical inefficiency and random error. These estimates are used to assess the efficiency level of each unit, providing decision-makers with information for improving production operations or policy responses. The SFA accounts for random error and inefficiency factors, making it more refined than a simple output/input ratio. The output/input ratio overlooks the distinction between random fluctuations and inefficiency in production, whereas the SFA separates the two, providing a more accurate estimate of efficiency. In the model, input variables (IR, PD, and 65Y) are the primary factors impacting the output (MR), while exogenous variables are controlling factors affecting mortality but are not directly used to measure relative efficiency across regions. The output or end result of interest in the model, is the COVID-19 MR.

In the SFA model, *V* represents the random error term, which describes the effect of exogenous factors and other random events on MRs. *U* is the inefficiency term, capturing the gap in efficiency due to the input variables. The *V* term is typically normally distributed, while the *U* term is non-negative, reflecting the level of inefficiency in each region. Efficiency and inefficiency are calculated based on the gap between the output variable (MR) and the efficiency frontier, not merely by the R-squared value. Efficiency scores are derived by comparing the theoretically most efficient MR to the actual MR for each region. This calculation accounts for both random error and inefficiency terms, rather than relying solely on model explanatory power. Although the infection rate is an input variable because it directly reflects the progression of the pandemic, the stringency index is an exogenous variable representing the strategies governments use to influence pandemic outcomes. Even if the infection rate perfectly explains the variation in mortality, efficiency would not necessarily be close to 1, as the SFA considers not only input variables but also inefficiency and random factors. The coefficient on the infection rate is important but is only part of the model and not the sole determinant of efficiency.

## Results

The two-phase SFA analysis conducted in this study utilised Maximum Likelihood Estimation to obtain the estimation results. The estimation outcomes of the first phase, pertaining to the year 2020, are presented in Table 3. Subsequently, the second-phase analysis encompassing the years 2021–2022 was carried out, wherein an additional exogenous variable, the cumulative FVR, was included. The estimation results for the second phase are delineated in Table 4. In Table 3, it is observed that during the initial year of the COVID-19 pandemic in 2020, both the infection rate and PD exhibited a significant negative correlation (estimates = −0.1088 and −0.0999, respectively) with the MR efficiency. This suggests that a decrease in the infection rate led to a reduction in the MR, while higher PD was associated with increased MRs. Furthermore, the proportion of the population aged 65 and over displayed a significant positive correlation (estimate = 0.2380) with MR efficiency, indicating that higher proportions of elderly individuals were linked to elevated MRs. This finding aligns with previous studies by Modig et al. [17] and Zhou et al. [27], which revealed that elderly COVID-19 patients experience higher MRs. Additionally, the MR, when factoring in the exogenous variable, exhibited a negative correlation with the inefficiency factor of SI (estimate = −5.1726), implying that more stringent enforcement of government control measures was associated with lower MRs.

**Table 3.** Estimates of the stochastic Frontier function of 2020.

Variable description	Coefficient	Estimate	Standard error	t-ratio
Constant	*β*_*0*_	0.9807**	0.3044	3.2214**
ln*IR*_*it*_	*β*_*1*_	−0.1088**	0.0350	−3.1062**
ln*PD*_*it*_	*β*_*2*_	−0.0999**	0.0346	−2.8872**
ln*65Y*_*jt*_	*β*_*3*_	0.2380*	0.0958	2.4835*
Constant	*δ*_*0*_	8.5624	8.4228	1.0166
ln*SI*_*jt*_	*δ*_*1*_	−5.1726	4.2547	−1.2157
ln*CDR*_*jt*_	*δ*_*2*_	−0.5458	0.6753	−0.8082
ln*DP*_*jt*_	*δ*_*3*_	0.9530	0.5453	1.7476
$\sigma_{s}^{2}=\sigma_{u}^{2}+\sigma_{v}^{2}$	$\sigma_{s}^{2}$	6.4341	4.4356	1.4505
$r=\sigma_{u}^{2}/\sigma_{s}^{2}$	*r*	0.9550	0.0360	26.5380
Log likelihood function = −182.29922				

1. ** and * represent significance at the 1% and 5% levels, respectively.

2. $\sigma_{u}^{2}$ and $\sigma_{v}^{2}$ represent the inefficiency error variance and random error variance, respectively; $\sigma_{s}^{2}$ is the total variance; *r* is the proportion of inefficiency error variance ($\sigma_{u}^{2}$) to total variance ($\sigma_{s}^{2}$).

**Table 4.** Estimates of the stochastic Frontier function of 2021–2022.

Variable description	Coefficient	Estimate	Standard error	t-ratio
Constant	*β*_*0*_	1.0049**	0.3026	3.3205**
ln*IR*_*it*_	*β*_*1*_	−0.3644**	0.0444	−8.2084**
ln*PD*_*it*_	*β*_*2*_	−0.1534**	0.0465	−3.2965**
ln*65Y*_*jt*_	*β*_*3*_	0.8500**	0.1264	6.7266**
Constant	*δ*_*0*_	8.6840**	0.7196	12.0674**
ln*SI*_*jt*_	*δ*_*1*_	−1.8792**	0.1734	−10.8393**
ln*CDR*_*jt*_	*δ*_*2*_	−0.0904	0.2094	−0.4316
ln*DP*_*jt*_	*δ*_*3*_	−0.3598*	0.1615	−2.2278*
ln*FVR*_*jt*_	*δ*_*4*_	0.2599*	0.1069	2.4311*
$\sigma_{s}^{2}=\sigma_{u}^{2}+\sigma_{v}^{2}$	$\sigma_{s}^{2}$	1.6878	0.1971	8.5640
$r=\sigma_{u}^{2}/\sigma_{s}^{2}$	*r*	0.9588	0.0314	30.5448
Log likelihood function = −484.40229				

1. ** and * represent significance at the 1% and 5% levels, respectively.

2. $\sigma_{u}^{2}$ and $\sigma_{v}^{2}$ represent the inefficiency error variance and random error variance, respectively; $\sigma_{s}^{2}$ is the total variance; *r* is the proportion of inefficiency error variance ($\sigma_{u}^{2}$) to total variance ($\sigma_{s}^{2}$).

Table 4 presents the correlations between various factors and the MR efficiency during 2021–2022. The COVID-19 pandemic infection rate and PD both show a significant negative correlation (estimates = −0.3644 and −0.1534, respectively) with MR efficiency. The proportion of the population aged 65 and above demonstrated a positive correlation (estimate = 0.8500) with MR efficiency, consistent with the findings from Phase 1. Furthermore, the MR, factoring in exogenous variables, displayed a negative correlation with the inefficiency factor of SI (estimate = −1.8792) and diabetes prevalence (estimate = −0.3598). Notably, the SI results mirrored those of 2020. These outcomes suggest that lowering the infection rate among diabetic patients could result in a reduction in MRs. In other words, individuals with diabetes exhibited a relatively higher MR following COVID-19 infection. This observation aligns with prior research indicating that older adults and individuals with underlying health conditions such as cardiovascular disease and diabetes, both at high risk for fatality, are at higher risk of severe COVID-19 [32,33]. Additionally, the MR displayed a positive correlation with the initial cumulative FVR inefficiency factor (estimate = 0.2599), suggesting that a higher initial FVR may contribute to an overall reduction in MRs. Hence, vaccination against COVID-19 is strongly recommended to mitigate mortality risks.

Table 5 presents data on the MR inefficiencies of 156 countries in combating the COVID-19 pandemic from 2020 to 2022. The top five countries in combating the pandemic in 2020, with their corresponding inefficiency values in parentheses, were Mongolia (0.0379), Singapore (0.0489), Bhutan (0.0829), Burundi (0.1084) and Cambodia (0.1136). Conversely, the bottom three were Yemen (0.8977), Mexico (0.8723) and Peru (0.8716). Notably, the average inefficiency value in 2020 stood at 0.62, indicating a significant deficiency in global efforts to combat COVID-19 mortality during the first year of the pandemic. This figure underscores substantial room for improvement, amounting to 62%. Detailed inefficiency values and efficiency rankings for 2021 and 2022 across other countries and regions worldwide are presented in Table 5.

**Table 5.** Estimates of the COVID-19 pandemic MR inefficiencies of SFA for 156 countries and regions.

No.	Location	Continent	Year						2020–2022 Mean of inefficiency value	2020–2022 Rank of efficiency
			2020 Inefficiency value	2020 Rank of efficiency	2021 Inefficiency value	2021 Rank of efficiency	2022 Inefficiency value	2022 Rank of efficiency		
1	Bhutan	Asia	0.08	3	0.02	4	0.01	19	0.04	1
2	Burundi	Africa	0.11	4	0.01	3	0.00	2	0.04	2
3	Mongolia	Asia	0.04	1	0.11	26	0.00	8	0.05	3
4	Singapore	Asia	0.05	2	0.10	23	0.03	65	0.06	4
5	Iceland	Europe	0.20	7	0.00	1	0.01	27	0.07	5
6	New Zealand	Oceania	0.40	23	0.01	2	0.02	63	0.14	6
7	Norway	Europe	0.37	17	0.03	6	0.03	86	0.14	7
8	Tajikistan	Asia	0.37	20	0.07	14	0.00	1	0.15	8
9	Belarus	Europe	0.36	15	0.10	24	0.02	38	0.16	9
10	Cyprus	Europe	0.35	13	0.09	21	0.03	91	0.16	10
11	Gabon	Africa	0.37	16	0.12	29	0.01	11	0.16	11
12	Seychelles	Africa	0.24	9	0.23	64	0.04	108	0.17	12
13	Central African	Africa	0.46	28	0.04	10	0.01	13	0.17	13
14	Denmark	Europe	0.43	26	0.05	11	0.03	79	0.17	14
15	Thailand	Asia	0.32	12	0.15	38	0.04	106	0.17	15
16	Botswana	Africa	0.17	6	0.35	94	0.03	64	0.18	16
17	Estonia	Europe	0.42	25	0.11	25	0.03	67	0.19	17
18	Côte d’Ivoire	Africa	0.37	19	0.18	48	0.02	40	0.19	18
19	Cambodia	Asia	0.11	5	0.46	115	0.00	7	0.19	19
20	Ghana	Africa	0.39	22	0.19	51	0.01	31	0.20	20
21	Japan	Asia	0.52	33	0.06	12	0.02	60	0.20	21
22	Guinea	Africa	0.37	18	0.21	57	0.02	58	0.20	22
23	Finland	Europe	0.53	34	0.03	7	0.06	128	0.21	23
24	Uruguay	South America	0.38	21	0.23	63	0.02	54	0.21	24
25	Papua New Guinea	Oceania	0.41	24	0.22	61	0.02	44	0.22	25
26	Qatar	Asia	0.23	8	0.40	103	0.02	53	0.22	26
27	Cuba	North America	0.50	31	0.15	39	0.00	5	0.22	27
28	Benin	Africa	0.58	43	0.08	17	0.00	3	0.22	28
29	Lao	Asia	0.56	41	0.08	16	0.03	81	0.22	29
30	Venezuela	South America	0.48	30	0.19	50	0.01	21	0.23	30
31	Mozambique	Africa	0.46	29	0.21	56	0.02	33	0.23	31
32	Malaysia	Asia	0.26	10	0.40	102	0.03	90	0.23	32
33	Australia	Oceania	0.66	70	0.02	5	0.02	59	0.24	33
34	Israel	Asia	0.54	35	0.14	35	0.04	93	0.24	34
35	Serbia	Europe	0.51	32	0.17	45	0.04	95	0.24	35
36	South Korea	Asia	0.59	48	0.09	20	0.04	107	0.24	36
37	UAE	Asia	0.35	14	0.35	96	0.02	51	0.24	37
38	Nicaragua	Central America	0.69	86	0.04	9	0.01	23	0.25	38
39	Netherlands	Europe	0.67	74	0.09	19	0.01	14	0.26	39
40	Switzerland	Europe	0.67	75	0.09	18	0.01	22	0.26	40
41	Brunei	Asia	0.62	57	0.12	31	0.03	72	0.26	41
42	Canada	North America	0.68	78	0.07	15	0.03	89	0.26	42
43	Sweden	Europe	0.68	84	0.07	13	0.03	76	0.26	43
44	Sri Lanka	Asia	0.27	11	0.49	121	0.04	98	0.27	44
45	Burkina Faso	Africa	0.55	38	0.23	66	0.03	87	0.27	45
46	Timor-Leste	Asia	0.62	56	0.18	49	0.01	30	0.27	46
47	Lithuania	Europe	0.58	44	0.21	55	0.03	71	0.27	47
48	Nigeria	Africa	0.65	69	0.16	42	0.01	12	0.27	48
49	China	Asia	0.77	134	0.03	8	0.01	26	0.27	49
50	Türkiye	Asia/Europe	0.55	37	0.24	73	0.03	83	0.27	50
51	Djibouti	Africa	0.55	36	0.28	80	0.00	4	0.28	51
52	Congo	Africa	0.60	51	0.23	65	0.01	15	0.28	52
53	Togo	Africa	0.68	82	0.15	37	0.02	48	0.28	53
54	Dominican	North America	0.68	81	0.17	44	0.01	10	0.29	54
55	Democratic	Africa	0.74	114	0.10	22	0.02	47	0.29	55
56	Mauritius	Africa	0.60	50	0.23	70	0.03	77	0.29	56
57	Portugal	Europe	0.66	72	0.17	47	0.03	70	0.29	57
58	Austria	Europe	0.70	92	0.15	40	0.02	55	0.29	58
59	Latvia	Europe	0.61	55	0.22	62	0.04	104	0.29	59
60	Sierra Leone	Africa	0.75	121	0.13	32	0.00	6	0.29	60
61	Ireland	Europe	0.74	112	0.12	27	0.03	75	0.29	61
62	Kazakhstan	Asia/Europe	0.58	46	0.30	84	0.01	16	0.30	62
63	Spain	Europe	0.75	124	0.12	30	0.03	85	0.30	63
64	France	Europe	0.75	122	0.14	34	0.02	57	0.30	64
65	Tanzania	Africa	0.70	91	0.20	53	0.02	39	0.31	65
66	Malta	Europe	0.71	97	0.14	36	0.07	141	0.31	66
67	USA	North America	0.67	76	0.21	54	0.05	115	0.31	67
68	Saudi Arabia	Asia	0.68	80	0.23	67	0.02	34	0.31	68
69	Cameroon	Africa	0.64	63	0.28	79	0.01	24	0.31	69
70	Chad	Africa	0.79	141	0.13	33	0.01	20	0.31	70
71	Ethiopia	Africa	0.65	67	0.27	76	0.02	41	0.31	71
72	Belgium	Europe	0.79	142	0.12	28	0.02	62	0.31	72
73	Albania	Europe	0.71	96	0.21	58	0.02	43	0.31	73
74	Czech	Europe	0.67	73	0.25	75	0.03	66	0.31	74
75	Niger	Africa	0.70	89	0.23	68	0.02	52	0.32	75
76	Morocco	Africa	0.69	88	0.24	72	0.02	46	0.32	76
77	Slovenia	Europe	0.74	113	0.16	43	0.05	118	0.32	77
78	Germany	Europe	0.75	123	0.17	46	0.03	74	0.32	78
79	Georgia	Asia/Europe	0.59	47	0.33	90	0.04	100	0.32	79
80	Barbados	North America	0.64	64	0.22	60	0.10	150	0.32	80
81	Madagascar	Africa	0.61	53	0.29	82	0.06	138	0.32	81
82	Costa Rica	Central America	0.63	60	0.30	85	0.04	103	0.32	82
83	Algeria	Africa	0.73	105	0.23	69	0.02	36	0.32	83
84	Cabo Verde	Africa	0.61	54	0.32	87	0.04	96	0.33	84
85	Croatia	Europe	0.67	77	0.24	74	0.06	131	0.33	85
86	Libya	Africa	0.62	58	0.33	88	0.03	73	0.33	86
87	Nepal	Asia	0.61	52	0.37	98	0.01	18	0.33	87
88	UK	Europe	0.80	144	0.16	41	0.03	82	0.33	88
89	Mauritania	Africa	0.70	90	0.27	78	0.03	80	0.33	89
90	Russian	Europe/Asia	0.62	59	0.33	89	0.05	117	0.33	90
91	Italy	Europe	0.79	143	0.19	52	0.04	101	0.34	91
92	Kuwait	Asia	0.55	39	0.47	117	0.01	25	0.34	92
93	Slovakia	Europe	0.58	45	0.41	105	0.05	121	0.34	93
94	Rwanda	Africa	0.59	49	0.42	109	0.02	61	0.35	94
95	Greece	Europe	0.76	130	0.22	59	0.06	134	0.35	95
96	Panama	Central America	0.70	93	0.31	86	0.03	84	0.35	96
97	Mali	Africa	0.76	131	0.27	77	0.03	68	0.35	97
98	Viet Nam	Asia	0.63	61	0.39	101	0.04	97	0.35	98
99	Argentina	South America	0.76	129	0.28	81	0.02	49	0.35	99
100	India	Asia	0.69	87	0.35	95	0.03	69	0.36	100
101	Angola	Africa	0.71	99	0.34	92	0.02	56	0.36	101
102	Zambia	Africa	0.66	71	0.42	108	0.02	50	0.37	102
103	Azerbaijan	Asia/Europe	0.65	68	0.40	104	0.05	127	0.37	103
104	Poland	Europe	0.72	104	0.35	93	0.05	120	0.37	104
105	Suriname	South America	0.64	65	0.43	110	0.04	113	0.37	105
106	Iraq	Asia	0.77	132	0.34	91	0.02	45	0.37	106
107	Pakistan	Asia	0.73	108	0.38	99	0.02	37	0.38	107
108	Chile	South America	0.75	120	0.29	83	0.10	151	0.38	108
109	Kenya	Africa	0.71	94	0.42	107	0.02	42	0.38	109
110	Lebanon	Asia	0.57	42	0.52	125	0.05	124	0.38	110
111	Fiji	Oceania	0.73	106	0.36	97	0.06	129	0.38	111
112	Namibia	Africa	0.44	27	0.67	139	0.05	123	0.39	112
113	Bangladesh	Asia	0.71	95	0.45	113	0.01	29	0.39	113
114	Haiti	North America	0.73	110	0.41	106	0.03	78	0.39	114
115	Senegal	Africa	0.71	100	0.47	119	0.01	28	0.40	115
116	Lesotho	Africa	0.73	107	0.47	118	0.02	35	0.40	116
117	Solomon Islands	Oceania	0.76	128	0.23	71	0.22	156	0.41	117
118	Romania	Europe	0.73	109	0.45	114	0.04	94	0.41	118
119	Liberia	Africa	0.81	147	0.44	111	0.01	17	0.42	119
120	Bolivia	South America	0.83	152	0.39	100	0.04	112	0.42	120
121	Hungary	Europe	0.76	127	0.44	112	0.06	135	0.42	121
122	Uganda	Africa	0.56	40	0.68	141	0.04	109	0.42	122
123	Jordan	Asia	0.68	83	0.55	129	0.04	110	0.42	123
124	Guyana	South America	0.72	102	0.50	123	0.06	139	0.43	124
125	El Salvador	Central America	0.78	140	0.49	122	0.04	92	0.44	125
126	Myanmar	Asia	0.72	101	0.60	134	0.00	9	0.44	126
127	Bahamas	North America	0.72	103	0.55	128	0.05	125	0.44	127
128	Bulgaria	Europe	0.77	136	0.48	120	0.07	142	0.44	128
129	Iran	Asia	0.82	150	0.47	116	0.05	116	0.44	129
130	Belize	Central America	0.74	116	0.55	127	0.05	126	0.45	130
131	Brazil	South America	0.75	119	0.56	131	0.04	114	0.45	131
132	Oman	Asia	0.65	66	0.69	143	0.02	32	0.45	132
133	Jamaica	North America	0.74	111	0.51	124	0.11	153	0.45	133
134	Colombia	South America	0.77	133	0.57	132	0.04	105	0.46	134
135	Bahrain	Asia	0.64	62	0.71	146	0.04	99	0.46	135
136	Philippines	Asia	0.74	115	0.55	130	0.09	149	0.46	136
137	Moldova	Europe	0.75	117	0.62	135	0.06	132	0.47	137
138	Zimbabwe	Africa	0.76	125	0.65	136	0.06	130	0.49	138
139	Malawi	Africa	0.76	126	0.66	138	0.06	140	0.49	139
140	Egypt	Africa	0.83	151	0.60	133	0.06	136	0.49	140
141	South Africa	Africa	0.77	135	0.65	137	0.06	137	0.50	141
142	Indonesia	Asia	0.78	137	0.68	142	0.05	122	0.50	142
143	Palestine	Asia	0.69	85	0.75	149	0.08	147	0.50	143
144	Sudan	Africa	0.82	149	0.54	126	0.16	154	0.51	144
145	Honduras	Central America	0.78	138	0.72	148	0.03	88	0.51	145
146	Paraguay	South America	0.71	98	0.75	150	0.08	148	0.52	146
147	Trinidad	North America	0.68	79	0.69	144	0.18	155	0.52	147
148	Tunisia	Africa	0.78	139	0.71	145	0.07	143	0.52	148
149	Gambia	Africa	0.81	146	0.71	147	0.05	119	0.52	149
150	Eswatini	Africa	0.75	118	0.78	152	0.06	133	0.53	150
151	Guatemala	Central America	0.81	148	0.67	140	0.11	152	0.53	151
152	Afghanistan	Asia	0.81	145	0.76	151	0.04	102	0.53	152
153	Ecuador	South America	0.85	153	0.79	153	0.04	111	0.56	153
154	Mexico	North America	0.87	155	0.82	154	0.08	144	0.59	154
155	Yemen	Asia	0.90	156	0.85	155	0.08	146	0.61	155
156	Peru	South America	0.87	154	0.89	156	0.08	145	0.61	156
	Mean		0.62		0.33		0.04		0.33	

Table 5 shows that the top 10 countries and regions with the best 3-year average efficiency rankings from 2020 to 2022, were, in order (inefficiency values in parentheses), Bhutan (0.0364), Burundi (0.0401), Mongolia (0.0520), Singapore (0.0591), Iceland (0.0744), New Zealand (0.1432), Norway (0.1433), Tajikistan (0.1487), Belarus (0.1595) and Cyprus (0.1597). Conversely, the bottom five were Peru (0.6149), Yemen (0.6083), Mexico (0.5901), Ecuador (0.5626) and Afghanistan (0.5347). Furthermore, Table 5 indicates that the average inefficiency values of MR across various regions worldwide in combating the COVID-19 pandemic were 0.62, 0.33 and 0.04 for the years 2020, 2021 and 2022, respectively. The overall average inefficiency value for these three years (2020–2022) was 0.33. This value suggests that while the effectiveness of countries worldwide in reducing COVID-19 mortality improved annually during the 3-year period, there remains a 33% room for enhancement overall.

This study revealed that countries or regions ranking highest in combating COVID-19 mortality over the 3-year period (2020–2022) consistently maintained excellent standings. Analysis of the research data highlighted Bhutan, Mongolia, Singapore, Iceland, and New Zealand as early adopters of stringent and comprehensive quarantine measures during the initial stages of the COVID-19 pandemic in 2020. Notably, the average annual SI for these five aforementioned countries exceeded 50 from 2020 to 2021. Furthermore, these nations implemented a national COVID-19 vaccination policy in 2021. With the exception of Mongolia, which attained a cumulative FVR of approximately 64%, the other four countries all surpassed this rate, reaching 75% or higher. Conversely, Yemen, ranking among the bottom three, exhibited a notably low SI (averaging around 25.5 over the three years) and achieved a cumulative FVR of only about 2.3% over the same period.

Additionally, Table 5 shows that the response capabilities of countries and regions worldwide to combat the pandemic improved each year over the 3-year period from 2020 to 2022. Among the major continents, Asia, Africa and Europe had similar representation in the top 20 countries and regions ranked highest in pandemic response during this period, while the majority of the lowest-ranked 20 countries and regions were in Africa and the Americas. When ranking the continents from best to worst based on average inefficiency values, the order was Oceania (0.28), Europe (0.29), Asia (0.32), Africa (0.33) and the Americas (0.40), with a gap of up to 0.12 in inefficiency values. Regarding economic development, developed countries demonstrated a stronger ability to confront the pandemic, with an inefficiency value of 0.26. By contrast, the average inefficiency value for the remaining countries and regions was 0.35, slightly above the overall average of 0.33.

## Discussion

This study uncovered several factors influencing COVID-19 mortality, including the infection rate, PD, the proportion of elderly individuals, the intensity of public health control measures, the presence of cardiovascular disease and diabetes, and the cumulative FVR. Notably, China implemented a zero-COVID policy in the early stages of the pandemic in 2020 [50,51]. Similarly, Singapore responded to the outbreak by establishing an inter-ministerial anti-pandemic working group in January 2020, implementing stringent zero-COVID policies, mask mandates, and rigorous border entry and exit tracking measures [52]. Likewise, Japan, Hong Kong, South Korea and Taiwan enforced universal mask mandates and stringent border control measures, effectively mitigating the spread of COVID-19. In the absence of an effective vaccine during the initial stage of the pandemic, the adoption of high-intensity public health control strategies indeed curbed the transmission of the virus, leading to reductions in both the infection and MRs. However, prolonged implementation of such stringent measures incurred significant losses at the national level, including economic, societal, and personal impacts, alongside decreased social interactions, impacting both physical and mental well-being [53].

Singapore has recognised the importance of swiftly acquiring and administering internationally certified vaccines to mitigate COVID-19 fatalities and curb transmission. With the rise in COVID-19 vaccination rates, coupled with an estimated 90% of the population having contracted the virus, Singapore attained a higher level of ‘hybrid immunity.’ In March 2022, Singaporean authorities announced further relaxation of social control measures, signalling the commencement of the final stage of the coexistence plan with COVID-19 [52]. These strategies and objectives in combating COVID-19, including the implementation of public health control measures to slow disease spread, sustaining healthcare system operations and promoting vaccination to mitigate severe illness and mortality, have yielded favourable outcomes [2]. Additionally, the findings of this study align with numerous previous studies indicating that individuals aged 65 and above, and those with chronic conditions such as cardiovascular disease and diabetes, may exhibit increased susceptibility to SARS-CoV-2 infection and higher MRs during the COVID-19 pandemic. Therefore, vaccination is strongly recommended for older adults aged 65 and above, as well as patients with chronic diseases, to mitigate infection and reduce mortality.

As time progresses, global COVID-19 infection cases and vaccination coverage continue to rise, prompting countries worldwide to gradually ease social restrictions and control measures. However, with the persistence of SARS-CoV-2 variants, there remains a risk of severe symptoms or even fatalities among the elderly and individuals with chronic conditions following infection. The key to global success in combating COVID-19 lies in coordinated efforts among nations to achieve low incidence rates and sustain moderate nonpharmaceutical interventions, alongside the implementation of adaptive social and economic policies [54–56].

### Limitations

This study has several limitations. First, this study is based on existing data and has undergone rigorous calculations; however, there remains a degree of uncertainty. Variability within the data may be influenced by factors such as sampling methods and model assumptions, potentially leading to a range of inherent errors. Second, the data source comprised data from official reports collected by the ‘Our World in Data’ team from various countries and regions. These data may be compromised due to differences in statistical methods, testing capabilities and estimation methods for COVID-19 across different regions. However, the data collection team is dedicated to minimising disparities and mitigating deviations between secondary data and real-world data. This ensures that the data can serve as a reliable source for informing social policies and public health measures. Third, this study primarily investigated the inefficiencies of social policies and public health measures implemented by countries in combating COVID-19 mortality during the pandemic. It did not delve into biological factors, the spread of virus variants, and virus-related mortality. Fourth, the use of SFA necessitates assumptions about the functional form before the estimation and allows for the analysis of only a single output item. Fifth, the factors influencing COVID-19 mortality extend beyond the three input items considered in this study, and the exogenous variables are not limited to the four variables examined here. Future research could explore additional or fewer exogenous variables. Sixth, there was indeed a discrepancy between the MR and the excess MR or the actual MR, resulting in slight differences in the data. Seventh, this study includes the lack of consideration of healthcare system differences across regions. Healthcare systems (such as resource allocation, ICU bed availability, and medical personnel numbers) significantly impact MRs, yet these factors are not included in the model. This may lead to an underestimation or overestimation of efficiency scores in some regions. Finally, the current research methods may be susceptible to certain statistical biases. Future studies could explore more effective research methodologies to replicate these findings.

## Conclusions

The empirical findings of this study reveal that the average inefficiency values of mortality during the global fight against the COVID-19 pandemic in 2020, 2021 and 2022 were 0.62, 0.33 and 0.04, respectively, with an overall average inefficiency value of 0.33. This suggests that while the effectiveness of reducing COVID-19 mortality is improved annually, there remains an average of 33% room for improvement. Despite the absence of effective vaccines in the early stages, the implementation of social distancing policies and public health control measures indeed reduced MRs. Additionally, higher PD, a larger proportion of the population aged 65 and over, and increased prevalence of diabetes were significantly associated with higher COVID-19 MRs. Furthermore, the study emphasises that increasing the cumulative FVR is the most effective method of reducing COVID-19 mortality. The COVID-19 pandemic exposed deficiencies in global public health policies and healthcare systems when confronting outbreaks of unknown infectious diseases. It is anticipated that the findings from this study will not only contribute to enhancing responses to and addressing the COVID-19 pandemic but also provide valuable insights for managing future pandemics of unknown viruses.

## Data Availability

This data can be found from ‘Our World in Data (2023)’.
